# Physician Order Entry Or Nurse Order Entry? Comparison of Two Implementation Strategies for a Computerized Order Entry System Aimed at Reducing Dosing Medication Errors

**DOI:** 10.2196/jmir.1284

**Published:** 2010-02-26

**Authors:** Alireza Kazemi, Uno GH Fors, Shahram Tofighi, Mesfin Tessma, Johan Ellenius

**Affiliations:** ^4^Research Centre for Strategic Studies on Health CareBaqyatallah University of Medical SciencesTehranIran; ^3^National Public Health Management Centre (NPMC)TabrizIran; ^2^Management Information Systems CentreHamadan University of Medical SciencesHamadanIran; ^1^Department of Learning, Informatics, Management and Ethics (LIME)Karolinska InstitutetStockholmSweden

**Keywords:** Medical order entry systems, decision support systems, clinical, medication erors, Iran, infant, newborn, patient safety

## Abstract

**Background:**

Despite the significant effect of computerized physician order entry (CPOE) in reducing nonintercepted medication errors among neonatal inpatients, only a minority of hospitals have successfully implemented such systems. Physicians' resistance and users' frustration seem to be two of the most important barriers. One solution might be to involve nurses in the order entry process to reduce physicians’ data entry workload and resistance. However, the effect of this collaborative order entry method in reducing medication errors should be compared with a strictly physician order entry method.

**Objective:**

To investigate whether a collaborative order entry method consisting of nurse order entry (NOE) followed by physician verification and countersignature is as effective as a strictly physician order entry (POE) method in reducing nonintercepted dose and frequency medication errors in the neonatal ward of an Iranian teaching hospital.

**Methods:**

A four-month prospective study was designed with two equal periods. During the first period POE was used and during the second period NOE was used. In both methods, a warning appeared when the dose or frequency of the prescribed medication was incorrect that suggested the appropriate dosage to the physicians. Physicians’ responses to the warnings were recorded in a database and subsequently analyzed. Relevant paper-based and electronic medical records were reviewed to increase credibility.

**Results:**

Medication prescribing for 158 neonates was studied. The rate of nonintercepted medication errors during the NOE period was 40% lower than during the POE period (rate ratio 0.60; 95% confidence interval [CI] .50, .71;*P* < .001). During the POE period, 80% of nonintercepted errors occurred at the prescription stage, while during the NOE period, 60% of nonintercepted errors occurred in that stage. Prescription errors decreased from 10.3% during the POE period to 4.6% during the NOE period (*P* < .001), and the number of warnings with which physicians complied increased from 44% to 68% respectively (*P* < .001). Meanwhile, transcription errors showed a nonsignificant increase from the POE period to the NOE period. The median error per patient was reduced from 2 during the POE period to 0 during the NOE period (*P* = .005). Underdose and curtailed and prolonged interval errors were significantly reduced from the POE period to the NOE period. The rate of nonintercepted overdose errors remained constant between the two periods. However, the severity of overdose errors was lower in the NOE period (*P* = .02).

**Conclusions:**

NOE can increase physicians' compliance with warnings and recommended dose and frequency and reduce nonintercepted medication dosing errors in the neonatal ward as effectively as POE or even better. In settings where there is major physician resistance to implementation of CPOE, and nurses are willing to participate in the order entry and are capable of doing so, NOE may be considered a beneficial alternative order entry method.

## Introduction

Medication errors can increase mortality and morbidity and add to healthcare costs [1]. Pediatric patients are at higher risk of medication errors because of weight-based dosing and difficulties in communicating with care providers [2]. Among all pediatric patients, neonates are the most vulnerable to medication errors because of their small body mass and extensive exposure to multiple medications in the neonatal ward or neonatal intensive care unit (NICU) [3]. Neonatal patients have special requirements, and during hospitalization, their weight and renal function may change frequently [4]. These changes demand frequent adjustment of prescription and administration dosages, which increases the risk of medication errors [5,6]. Dosing errors are the most prevalent type of errors in neonates, and most of these occur at the time of prescription [7]. Antibiotics are the most frequently prescribed type of drug involved in neonatal dosing errors [7,8]. Also reported have been severe adverse events due to miscalculated doses of anticonvulsants [9]. Therefore, strategies to prevent dosing errors of antibiotics and anticonvulsants in neonates should be prioritized.

In previous studies, computerized physician order entry (CPOE) with decision support functionalities has reduced dosing errors of antibiotics among inpatient neonates [10,11]. Despite promising results, only about 2% to 20% of the hospitals in high-income countries have successfully implemented CPOE [12]. Among several barriers to implementation, high implementation costs, physician resistance, and user frustration have been found to be the most important [13-15]. In many hospitals' order entry systems, nurses or other nonphysician health personnel enter medical orders into the computer [16]. Even in hospitals that have successfully implemented strictly physician order entry (POE), some orders are entered by the nurses [17]. Some investigations have shown that nurses often have more positive attitudes toward computerized systems than physicians [18]. Therefore, the involvement of nurses in the order entry process may increase the rate of success and reduce physicians' resistance [16,17]. In a number of recent studies, researchers have defined CPOE as computerized provider order entry that includes participation by credentialed nurses [19].

The successful implementation of POE becomes even more complicated in middle- and low-income countries with economic and human resource constraints [20]. One such country is the Islamic republic of Iran, a country in the Middle East with a population of 70 million as of 2006 [20,21]. Iran is cooperating with the World Health Organization to extend the use of information technology and evidence-based decision making in the health sector [22].

Studies performed in Iran demonstrate that medication dosing errors and adverse drug events (ADE) are significant problems for the Iranian healthcare system [23,24]. In almost all Iranian hospitals that have implemented electronic medical record systems, nurses or professional operators enter medical information into the computer. Physicians do not interact with the system at all, or their interaction is limited [20].

In 2007, a POE system was implemented in the neonatal ward of an Iranian teaching hospital. The aim of this project was to investigate whether the implementation of the system reduced medication errors and to investigate transferability of the system to other wards of this hospital as well as to other teaching hospitals in Iran [20]. The introduction of the system was found to reduce medication errors of antibiotics and anticonvulsants [25]. However, the busy residents were reluctant to enter all prescribed orders into the computer. After several interview sessions with attending physicians, residents, and nurses, a new implementation model was introduced to address this challenge.

In the new order entry model, nurses entered the orders into the computer, and the resident physicians verified the correctness of the orders and countersigned them electronically. Despite the successful implementation of this method, its effectiveness in reducing medication errors still needed to be examined.

The aim of this study was thus to determine whether the new collaborative order entry method was as effective as the strictly physician order entry method in reducing nonintercepted dose and frequency medication errors of antibiotics and anticonvulsants.

## Methods

### Setting

The study was conducted in the neonatal ward of a 400-bed tertiary care referral teaching hospital (Besat) in the capital city of Hamadan that provides a variety of clinical services. Hamadan is a province in the northwest of Iran with almost 1,700,000 inhabitants. Besat's neonatal ward is a 17-bed clinical ward that includes two NICU beds.

### System description

#### Hospital Information System (HIS)

Sayan-HIS (Sayan Rayan Co Ltd, Hamadan, Iran) is a commercial patient-centered hospital information system (HIS) that is used in all fifteen university-affiliated hospitals in Hamadan. It is a client-server application that uses MS-SQL server 2003 as its database. Users interact with the system in a local area network and through desktop computers installed at workstations. The system includes an administrative as well as a clinical information system. The administrative information system handles patient billing and the insurance company interface as well as providing various reports for the financial controllers and management.

#### Clinical Information System

The clinical information system of Sayan-HIS includes an order-entry based prescription system. When the physician’s orders are entered into the computer, the prescription system delivers the requested orders for medications, lab tests, and imaging to the relevant hospital sections at the appropriate time. The system limits the selection of drugs and their pharmaceutical forms (vial, ampoule, tablet, etc) through drop-down lists and preconstructed orders. The system was functional and routinely used with all explained features in all wards of the Besat hospital at the time of this study. The system also includes a rule-based clinical decision support system that is capable of alerting and correcting an erroneously prescribed dose or frequency of an antibiotic or anticonvulsant for neonatal patients.

#### Clinical Decision Support System (CDSS)

The dose and frequency decision support system was developed in 2007. The knowledge base was completed for all routine antibiotics and anticonvulsants by using the local guidelines of best practice based on pediatric reference books approved by the National Board of Pediatrics in Iran [26-29]. Prescription decision criteria were based on each patient’s clinical diagnosis, age, weight, gestational age, and estimated glomerular filtration rate (GFR). Three neonatal specialists and one pediatric nephrologist reviewed and approved the CDSS calculation methods.

**Figure 1:**
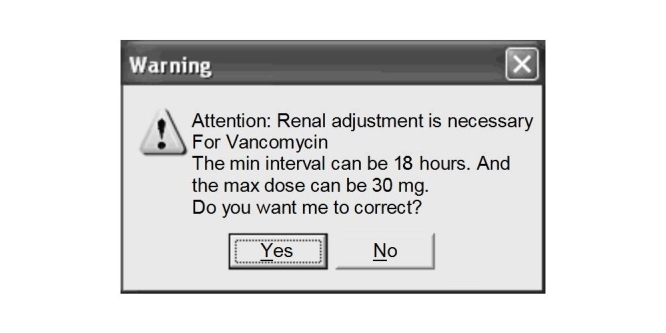
A warning message for dose and frequency errors that gives the reason for the warning (Note that the figure shows a translated mockup)

The system displayed warning messages on the prescription page whenever it detected a dose or frequency medication error based on the previously mentioned criteria (Figure 1). The warning supplied the appropriate dose and/or frequency as well as an explanation as to why the warning had appeared. The prescriber was then allowed to comply with the warning's recommended dosage or to ignore it. The responses of the prescribers to the warnings were recorded by the system in an error registration table. A detailed description of the CDSS and its interactions with the prescription system was presented in a previous paper [25].

### Inclusion criteria and study population

The study population consisted of neonatal patients who were prescribed antibiotics for infectious diseases or anticonvulsants for seizure and who received at least one dose of these drugs. All orders for antibiotics and anticonvulsants for these patients were included.

### Definition of medication errors

Normal ranges of doses and frequencies of the selected medications were calculated based on the published references cited above [26-29]. Medication errors for the purpose of this study were defined as overdoses or underdoses or curtailed or prolonged intervals.

In this study, we focused on both prescription and transcription errors but not on administration errors. A prescription error was defined as an error that occurred during the prescription stage. The prescription stage included errors in orders written initially on paper or directly entered into the computer by providers, or when a dose should have been changed but the prescriber ignored the computer warning or neglected to correct the prescription. The latter case mostly occurred when a dose decision criteria (age group, weight, GFR, etc) was changed during the hospitalization period.

A transcription error was defined as an error that occurred after the prescription stage. This type of error could have happened when information was transferred from handwritten orders to the computer or vice versa. This type of error could also have happened during registration of the doses in the paper-based medication administration chart or in the paper-based Kardex kept at the nurses’ station that contains each patient’s scheduled medications. A medication order with errors in both the prescription and the transcription stage was considered a prescription error.

Medication errors that did not reach patients were categorized as intercepted errors, and medication errors that reached patients were categorized as nonintercepted errors. Interception of medication errors could have occurred during two different phases in the prescription process. The first phase was when the physician prescribed an erroneous dosage but the CDSS corrected the error following a warning with which the physician complied. The second phase was when an erroneous dosage was detected and corrected by the nurse or physician before the medication was administered to the patient.

Nonintercepted medication errors could have occurred during the prescription phase or during the transcription phase. Such errors could have happened if the prescriber registered medications erroneously into the paper-based order or directly into the computer or if the paper-based order was erroneously transcribed into the system or Kardex by the nurse.

### Data collection and review process

This study was a prospective study. Physicians' responses to the warnings were stored in a table together with both the erroneous and the corrected doses and frequencies. Therefore, it was possible to detect prescriptions that were initially incorrect but were intercepted by the warnings. In addition, one of the authors (AK) reviewed all relevant paper-based medical documents and electronic patient records. This included handwritten orders during the NOE period, electronic orders of both periods, and paper-based and electronic medication administration charts of both periods (Table 1).

**Figure 2:**
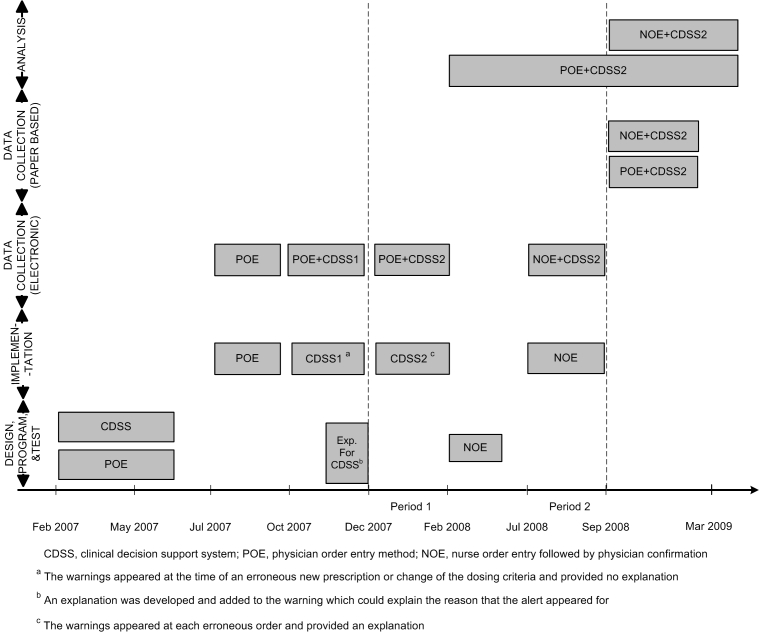
Development, implementation, and evaluation of clinical decision support system (CDSS), physician order entry (POE), and nurse order entry followed by physician confirmation (NOE) in the neonatal ward of the Besat hospital

Electronic orders and the electronic medication administration chart were tabulated automatically in the system's database during each period. However, data collection of the paper-based medication administration chart during both periods and the paper-based orders during the NOE period was performed after the NOE period (Figure 2). Analyses were performed after the completion of each period.

By triangulating different sources of data we could detect those medications that were prescribed erroneously and were not intercepted by the warnings, but were intercepted by physicians or nurses before the medications were administered to the neonates. In these cases, the electronic orders were registered with erroneous doses, but the paper-based medication administration charts were registered with the correct doses.

As well, medications that were prescribed erroneously and also registered in paper-based medication administration charts with erroneous doses were considered nonintercepted prescription errors. Medications that were prescribed with correct dosages but were registered in the paper-based medication administration chart with erroneous doses or frequencies were considered nonintercepted transcription errors. This last type of error could have occurred because of frequent transcriptions between paper-based and electronic orders, orders and the nursing Kardex, or the Kardex and paper-based medication administration charts.

### Measuring medication errors

The rate of nonintercepted medication errors was calculated using the following four measures.

*Patient-day* was defined as one day of hospitalization for a patient who received medication therapy during the day. If all medications in all prescribed orders on the same day were correct, that day was as one correct patient-day, otherwise it was counted as an erroneous patient-day.

*Medication-day* was defined as a medication that was prescribed and continued for a patient on the same day. If all prescribed orders of a medication on the same day were correct, it was counted as one correct medication-day, otherwise it was counted as an erroneous medication-day.

*Order* was defined as a collection of prescribed medications, lab tests, imaging, and so on written by a physician for a patient during or after a visit to the patient’s bedside. If all prescribed medications in the same order were correct, it was counted as one correct order, otherwise it was counted as an erroneous order.

*Ordered medication* was defined as a medication prescribed in an order. If the prescribed medication was correct, it was counted as one correct ordered medication, otherwise it was counted as an erroneous ordered medication.

### Study periods and their characteristics

This study was designed to compare two medication order entry methods each of which was studied over a 2-month periods. During period 1, or POE, physician order entry was followed by nurse verification and countersignature; during period 2, or NOE, nurse order entry was followed by physician verification and countersignature. The study was conducted between December 2007 and September 2008 (Table 1).

**Table 1:** Computerized order entry periods at the neonatal ward of the Besat hospital

	Period 1: Dec 2007 - Feb 2008	Period 2: Jul - Sep 2008
Intervention	POE^a^	NOE^b^
Order entry	Resident physicians	Nurses
Verification and countersignature	Nurses	Resident physicians
CDSS^c^ functionality	Warnings	Warnings
When warnings displayed	Order entry	Countersignature
Documentation	E-Prints^d^	HWO^e^+E-Prints^d^
Review process	EO^f^+PBMAC^g^+EMAC^h^+ERT^i^	HWO^e^+EO^f^+PBMAC^g^+EMAC^h^+ERT^i^

^a^ Physician order entry

^b^ Nurse order entry

^c^ Clinical decision support system

^d^ Electronic prints of prescriptions

^e^ Handwritten orders

^f^ Electronic orders

^g^ Paper-based medication administration chart

^h^ Electronic medication administration chart

^i^ Error registration table

#### Period 1: Physician Order Entry Followed by Nurse Verification and Countersignature (POE)

**Figure 3:**
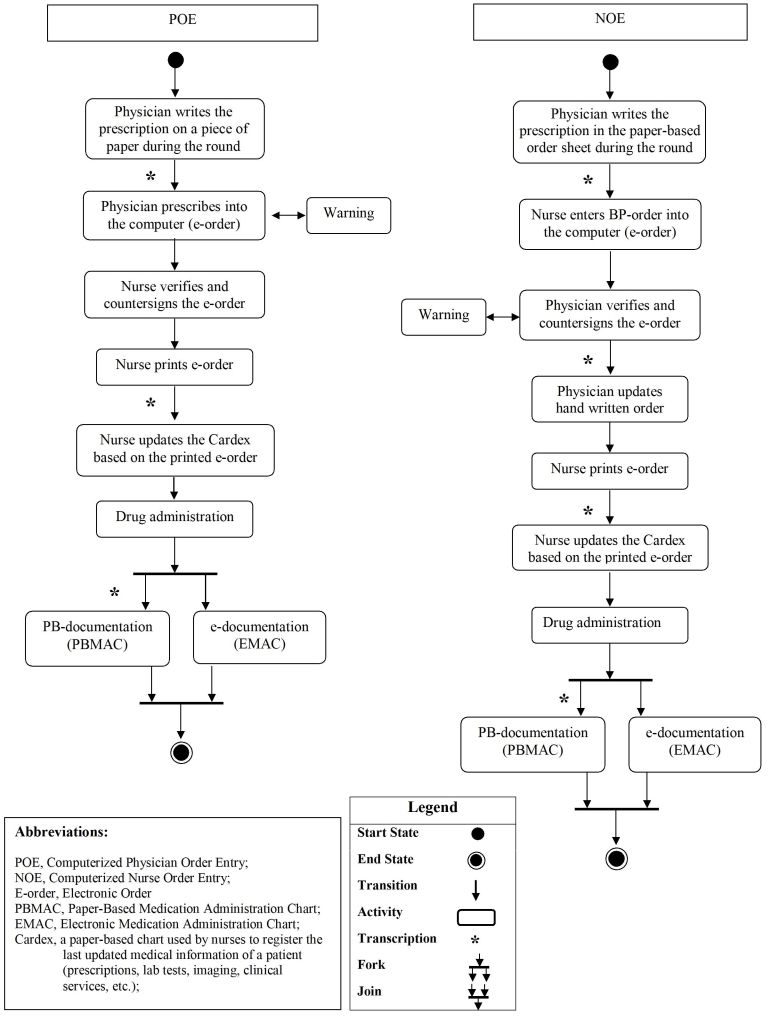
Medication prescription and administration workflows during the POE and NOE periods in the neonatal ward of the Besat hospital

During period 1, resident physicians entered all prescription orders directly into the computer and paper-based orders were eliminated (Figure 3). To reduce possible data entry errors, a nurse verified and countersigned each electronic order that physicians had entered into the computer. This verification was designed to reduce the likelihood of making typographical errors or of selecting incorrect drugs from the drop-down menus. A further design consideration was to remind physicians about obvious dosing errors of those medications that were not included in the knowledge base (ie, drug groups other than antibiotics and anticonvulsants) and consequently warnings could not help to prevent them. Because Iranian law does not permit electronic signatures, each electronic order was printed and saved in the patient's medical file after it was countersigned [30] (Table 1).

Also in this period, each prescription line was assessed by the decision support system as it was prescribed by the resident physician. When a resident had ignored a warning, the ignored warning appeared each time the resident renewed the order with the same erroneous dose and frequency, or when the resident prescribed a new dosage that was also erroneous (Figure 2).

The design, programming, and testing of the decision support system for the POE method started in February 2007 (Figure 2). During this period, the functionality of the CDSS was gradually developed [25]. During period 1 of the current study, the frequency and format of the displayed warnings had been optimized, and these remained unchanged in period 2. Therefore, this period of POE was selected to be compared with NOE.

#### Period 2: Nurse Order Entry Followed by Physician Verification and Countersignature (NOE)

During period 2, the care providers of period 1 switched their roles in order entry and countersignature, vis-à-vis (Table 1 and Figure 3). Resident physicians wrote the initial orders on the prescription papers and delivered them to the nurses who subsequently entered them into the computer. The residents then verified and countersigned the orders electronically. Warnings appeared only at the time of physicians’ countersignatures. Therefore, in this new model, warnings appeared to the residents but not to the nurses (Figure 3). This strategy was adopted because in a previous study of CPOE in Iran, physicians were reluctant to let their errors be disclosed to nurses and wished to receive the warnings themselves [20]. However, after the implementation of POE, the residents started to resist performing the order entry because they perceived it to be very time consuming. However, they still wanted to receive the warnings themselves without allowing the nurses to see them. The new model was designed in close collaboration with the involved physicians and nurses to address this issue.

After the physician's verification and countersignature, the electronic prescription was printed, and if a warning had been complied with that led to a change of dose or frequency, both the nursing Kardex and the patient file were updated (Figure 3). In period 2, both electronic prints and handwritten prescription papers were saved in the patient's file.

### Statistics

Descriptive statistical analysis was used to determine medians for continuous variables and percentages for categorical variables. The median and the 25^th^ and 75^th^ percentiles of medication errors and the medians of age at admission, gestational age, and length of hospital stay were computed. Chi-square tests were performed for nonordinal categorical variables. For continuous variables, the nonparametric Mann-Whitney tests were employed to determine differences in the median error rates between the POE and NOE periods when there was remarkable deviation from normality. Rates of errors were reported pertaining to orders, ordered medications, medication days, and patient days. Error rate differences between the POE and NOE periods were calculated as *d* = absolute value of the error rate during the POE period minus the error rate during the NOE period. Rate ratio (RR) was defined as the rate of errors during the NOE period divided by the rate of errors during the POE period. RR < 1 indicates that NOE has a "protective effect," and RR > 1 demonstrates that NOE has an "incremental effect" for medication errors. Confidence intervals for the ratios were determined under the assumption that the number of events per 100 patient-days followed a Poisson distribution. Miettinen’s test-based approximation was used to calculate the confidence interval for the rate ratios. The level of statistical significance was specified at 0.05. Statistical analyses were performed using SPSS version 17 (SPSS Inc, Chicago, IL, USA). EPI Info version 6.0 (Centers for Disease Control and Prevention, Atlanta, GA, USA) was used to calculate chi-square for trend Mantel extension test [31] to examine an increasing or decreasing linear trend in the severity of overdose errors during the NOE period compared with the POE period.

### Ethical considerations

The National Ethical Committee of the Ministry of Health and Medical Education in Iran granted permission for this study in 2005. All physicians and nurses who participated volunteered to take part in the study, and a verbal informed consent was obtained and tape recorded.

## Results

A total of 158 neonates were included in this study (Table 2). No significant differences were observed in the distribution of sex, age at admission, or gestational age of these neonates between the POE and NOE periods.

**Table 2:** Distribution of the characteristics of patients included in the study, numbers of orders, and numbers of medications in the two study periods, POE and NOE

	POE	NOE
Patients	69	89
Male/female	35/34	41/48
Median age at admission (days)	7	5
Median gestational age (weeks)	38	38
Orders	972	978
Ordered medications^a^	2357	2297
Patient days^b^	601	648
Medication days ^c^	1466	1492
Median length of hospital stay (days)	9.1	6.7

^a^ A prescribed medication in an order is one ordered medication

^b^The number of days that patients received antibiotics or anticonvulsants

^c^The number of days that included medications were continued for patients

Medication errors were reduced to an equal extent during both the POE and NOE periods (Table 3). However, as the rate of errors that were intercepted by the warnings increased from 4.5% in the POE period to 8.1% in the NOE period (rate ratio 1.80, 95% confidence interval [CI] 1.43, 2.27; *P* < .001), the rate of nonintercepted errors dropped from 12.8% to 7.6% respectively (rate ratio 0.60, 95% CI 0.50, 0.71; *P* < .001). Most of the intercepted errors were caught by the warnings at the prescription stage; only a few errors were subsequently detected and intercepted by nurses or physicians before they were administered to the patients. The number of errors that were intercepted by the care providers was not significantly different between the two periods.

**Table 3:** Intercepted and nonintercepted medication errors and their rate ratio in the POE and NOE periods

Type of medication error	POE (n^a^=2357)	NOE (n^a^=2297)	RR^b^ (95% CI^c^)
Intercepted by the warnings	106 (4.5)^d^	186 (8.1)	1.80 (1.43, 2.27)^e^
Intercepted by care providers^f^	12 (0.5)	11 (0.5)	0.94 (0.42, 2.13)
Nonintercepted	301 (12.8)	175 (7.6)	0.60 (0.50, 0.71)^e^
Total	419 (17.8)	372 (16.2)	0.91 (0.8, 1.03)

^a^ n = number of ordered medications

^b^ Rate ratio

^c^ Confidence interval

^d^ Numbers in parentheses are percentages of errors calculated as [(number of errors)/ n] * 100

^e^ *P* < .001

^f^ Includes errors intercepted by nurses or physicians after the prescription stage and before the administration

Table 4 depicts different measurement units employed to calculate the rate and rate ratios of nonintercepted medication errors following the implementation of NOE in contrast to POE period. All measurements showed a highly significant reduction of medication errors from the POE period to the NOE period. However, the highest rate difference (9.5%) was seen when calculated according to patient days (rate ratio 0.61; 95% CI 0.49, 0.77; *P* < .001), and the lowest (5.2%) when using the ordered medications method (rate ratio 0.60; 95% CI 0.50, 0.71; *P* < .001). NOE showed a greater reduction effect on medication errors in all four calculation methods.

The median nonintercepted error per patient decreased from 2 (25th percentile = 0 and 75th percentile = 5) in the POE period to 0 (25th percentile = 0 and 75th percentile = 2) in the NOE period (*P* = .005). In the POE period, about 38% (26/69) of the patients did not experience any dosing errors, while in the NOE period, about 53% (47/89) of them were error-free (the rate difference was 15%).

**Table 4:** Rates and rate ratios of nonintercepted medication errors in POE and NOE using different measurements

Measurement unit	POE Errors/n (%)^a^	NOE Errors/n (%)^a^	RR^b^ (95% CI^c^)
Orders	221/972 (22.7)	142/978 (14.5)	0.64 (0.53, 0.77)^d^
Ordered medications	301/2357 (12.8)	175/2297 (7.6)	0.60 (0.50, 0.71)^d^
Medication-days	211/1466 (14.4)	129/1492 (8.6)	0.60 (0.49, 0.74)^d^
Patient-days	147/601 (24.5)	97/648 (15.0)	0.61 (0.49, 0.77)^d^

^a^ errors is the number of errors per measurement unit; n is the number of measurement units (see Table 2); the number in parentheses is percentage of errors calculated as [(number of errors)/ n] * 100

^b^ Rate ratio

^c^Confidence interval

^d^*P* < .001

**Figure 4:**
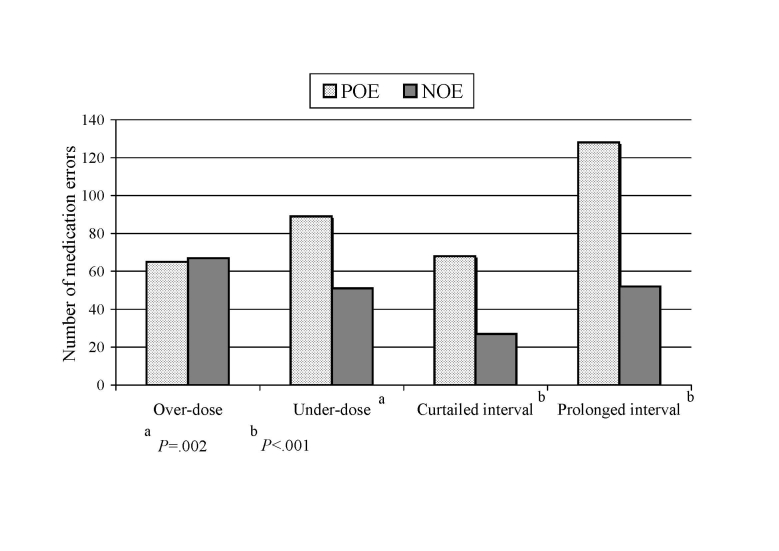
Subtypes of nonintercepted dose and frequency medication errors

We divided nonintercepted medication errors into overdose, underdose, curtailed interval, and prolonged interval. We found that all subtypes of errors except overdose errors decreased significantly from the POE to the NOE period (*P* = .002 for underdose, and *P* < .001 for curtailed and prolonged interval errors) (Figure 4). The rate of overdose errors remained unchanged. However, there was a linear decreasing trend in severity of the overdose errors in the NOE period compared with the POE period (chi square for trend = 5.2; *P* = .02). The maximum registered overdose was less than 250% of the normal dose in the NOE period and less than 300% in the POE period. Two-fold or greater dosing errors occurred in about 25% (16/65) of overdosed medications in the POE period, while this occurred only in about 7% (5/67) of overdosed medications in the NOE period (*P* = .007).

The rate of prescription errors decreased significantly from 10.3% in the POE period to 4.6% in the NOE period (rate ratio 0.45; 95% CI 0.36, 0.56; *P* < .001) (Table 5). Meanwhile, transcription errors showed a slight increase from 2.5% in the POE period to 3% in the NOE period. However, in both the POE and NOE periods, the majority of nonintercepted errors occurred in the prescription phase (80% in the POE and 60% in the NOE period). Therefore, the overall rate of errors also decreased from the POE period to the NOE period (rate ratio 0.60; 95% CI 0.50, 0.71; *P* < .001).

**Table 5:** Nonintercepted prescription and transcription errors in the ordered medications of POE and NOE and their rate ratio

Error type	POE (n^a^ = 2357)	NOE (n^a^ = 2297)	RR^b^ (95% CI^c^)
Prescription errors	242 (10.3)^d^	106 (4.6)	0.45 (0.36, 0.56)^e^
Transcription errors	59 (2.5)	69 (3.0)	1.20 (0.85, 1.69)
Total	301 (12.8)	175 (7.6)	0.60 (0.50, 0.71)^e^

^a^ n = number of ordered medications

^b^ Rate ratio

^c^Confidence interval

^d^Numbers in parentheses are percentages of errors calculated as [(number of errors)/ n] * 100

^e^ *P* < .001

Many prescription errors occurred because the prescriber set an erroneous dose at the time of prescription. Other errors occurred when one or more of the dose decision criteria (age, weight, GFR, etc) had changed since the last prescription but the prescriber did not change the prescribed order and repeated the previously ordered dose and frequency (Table 6). In the NOE period, many transcription errors occurred when the electronic order was updated following a warning with which the prescriber had been complied, but the paper-based order was not updated or was updated with a different dose or frequency. This type of error did not happen in the POE period since the handwritten orders were eliminated in this period. The number of errors that occurred following incorrect registration of the paper-based medication administration chart although the electronic medication administration chart was correct, did not significantly differ between the POE and NOE periods. The rate of errors that occurred because the prescriber neglected to update the paper-based Kardex was approximately the same during the NOE and POE periods.

The total number of warnings was 312 in the POE period and 339 in the NOE period. The number of warnings with which the prescribers complied increased significantly from 44% (136/312) in the POE period to 68% (232/339) in the NOE period (*P* < .001).

**Table 6:** Distribution of nonintercepted medication errors at different registration steps of the POE and NOE periods

Reasons for dose and frequency errors	P/T^a^	POE (n^b^=2357)	NOE (n^b^=2297)	RR^c^ (95% CI^d^)
Ordered dosage was initially incorrect	P	163 (6.9)^e^	70 (3.0)	0.44 (0.34, 0.58)^f^
Order continued with the previous dose despite the change in dosing criteria	P	79 (3.4)	36 (1.5)	0.47 (0.32, 0.69)^f^
PB-order inconsistent with E-order	T	0 (0.0)	22 (1.0)	N/A^f^
PBMAC inconsistent with EMAC^g^	T	24 (1.0)	13 (0.6)	0.56 (0.28, 1.09)
Prescribed order changed but still the previous dose administered (Kardex was not updated)	T	35 (1.5)	34 (1.5)	1.00 (0.62, 1.59)
Total		301 (12.8)	175 (7.6)	0.60 (0.50, 0.71)^f^

^a^ P/T: prescription or transcription error

^b^ n = number of ordered medications

^c^ Rate ratio

^d^ Confidence interval

^e^Numbers in parentheses are percentages of errors calculated as [(number of errors)/ n] * 100

^f^*P* < .001

^g^PBMAC, paper-based medication administration chart; EMAC, electronic medication administration chart

## Discussion

Previous studies have highlighted a low compliance with POE and a high resistance to acceptance of it among physicians, as well as the failure of POE systems in developed countries [12-14,32]. The initial intention of this study was to investigate whether NOE as an alternative order entry method was at least as effective as POE in reducing medication dosing errors. Surprisingly, we observed that the overall rate of nonintercepted dose and frequency medication errors was in fact lower under NOE than POE.

One reason for the lower error rate is that the prescribers complied with a higher rate of warnings in the NOE than in the POE period. The result was a significant reduction in the rate of nonintercepted prescription errors. Other studies have also reported that decision support systems can reduce prescription errors if prescribers comply with the system's recommendations [33,34]. Since in the POE period a majority of the nonintercepted errors occurred in the prescription stage, reduction of prescription errors resulted in an overall reduction of nonintercepted errors. Previous studies in the pediatrics and neonatal settings show that a majority of errors occur in the prescription stage [7].

In addition, most of the errors that were not intercepted by the warnings in the prescription stage reached the patients. Only a few of these errors were caught by the care providers. This reveals the importance of the dose decision support system and prescribers' compliance with the system's recommendations in this context. In developed countries, in addition to decision support systems, clinical pharmacists in many hospitals interact with care providers and supervise the preparation and administration of medications. In many cases, the pharmacy department is responsible for preparing ready-to-administer doses. The results of two studies, one in the United States and one in the United Kingdom, demonstrated a 66% to 80% reduction of medication errors following the active involvement of a senior clinical pharmacist in the clinical rounds [35,36]. However, in most hospitals in Iran, pharmacists and clinical pharmacologists do not participate in clinical rounds. The pharmacy does not prepare ready-to-administer doses; nurses in the wards are responsible for these. In Iranian hospitals, many responsibilities are left to the nurses. This is mostly because a very hierarchical system exists in these hospitals [20]. Hospital managers often assign to nurses, who are at the bottom of this hierarchy, tasks that physicians or pharmacists object to performing [20]. Medical data entry is one of these tasks. In Iranian hospitals, there are few legal or administrative incentives for physicians to enter medical data into electronic systems [20,37]. Therefore, strategies such as NOE, which require less physician time, may increase physicians’ compliance and result in a more sustainable implementation of computerized provider order entry systems.

In addition, there are several other possible explanations for increased compliance in the NOE period. One explanation is that in the strictly physician order entry period, resident physicians were more likely to have focused on data entry than on the warnings. They may have ignored the warnings unintentionally because of frustration and stress following a prolonged data entry session. A previous study showed that it is difficult to successfully implement systems that physicians consider to be time consuming [14]. The authors stated that prolonged data entry and user frustration were important causes of the failure of CPOE in their study [14]. However, in the nurse order entry method, physicians needed only to focus on prescription errors and warnings. This could have increased their attention to the displayed warnings and resulted in better compliance. It is also possible that the new collaborative environment in the NOE period created a better understanding of the advantages of the CDSS and resulted in better physician compliance with the system's recommendations. Today, more and more hospitals in western countries are attempting to redefine traditional borders between doctors and nurses by creating closer collaboration between them in all clinical activities [38,39]. In countries like Iran, where a hierarchical and physician-centered atmosphere exists in clinical settings [20], for CPOE systems to be successful, it is important that managers and policy makers create a collaborative and patient-centered climate.

Another possible explanation for higher compliance in the NOE period, is that NOE was designed in close collaboration with care providers and reflected their opinions. Therefore, care providers were more compliant with the new order entry method. As other studies have emphasized, care providers’ acceptance and their collaboration in the development process are key factors in successful implementation of computerized order entry systems [17].

In addition, the reduction in medication errors can also be attributed to the fact that prescription orders may have been double-checked by the prescribing physicians in the NOE period. In the NOE model, prescribers had to check transcribed orders before signing them. This provided them with the possibility of double-checking what they had already prescribed before they received any warnings. This double-checking, independent of CDSS warnings, can also explain the observed reduction in prescription error rates in the NOE period.

In our study, the increase of transcription errors from the POE to the NOE period was small. Considering the workflow of the two order entry methods (Figure 3), NOE seems to be more complex than POE. Therefore, the rate of transcription errors should be higher in the NOE model than the POE model. However, since Iranian law prevents elimination of paper-based medical records [30], any medical order entry system, even POE, includes redundant recordings and documentation. Therefore, in such a context, POE has no apparent advantage over NOE in terms of transcription errors. In the United States and some European countries, where computerized order entry has reduced paperwork, POE has become a powerful tool to prevent transcription errors [33,40-42].

In our study, despite the nonsignificant difference in the overall rate of transcription errors between the POE and NOE periods, there are certain types of these errors that could be eliminated by POE. When a physician directly prescribes into the computer and prints the order, there can be no discrepancy between the electronic and paper-based order. In contrast, when using NOE, a physician must write a paper-based order and sign it for the nurse so that the nurse can enter the order into the computer. Since this paper-based order is a legal document, when a warning has been accepted, the resident must also update the paper-based order; negligence may result in nonintercepted transcription errors as in our study. Other types of transcription errors were not significantly different between the POE and NOE periods because after the prescription stage, the transcription and administration flows are the same in both systems.

In order to reduce transcription errors in Iran, prescription workflow should be simplified, and paper work should be limited. These strategies can save time, reduce costs, and may directly affect care providers' satisfaction resulting in higher acceptance. In Iran, many care providers complain that paperwork has dominated clinical care and that computerized systems have created many redundant registrations and documentation [20]. However, adapting Iranian law to demands from the digitized world is a challenge.

### Methodological considerations

In this study, we calculated the number of nonintercepted medication errors using four measurement methods. Previous studies have used one or more of these measurement methods to report medication errors [43,44]. However, the calculation method affects the error rate considerably. For example, in our study, medication error rates reported per patient-day were twice the error rates reported per ordered medication. The reason is that in the patient-day method, several medications in several orders on the same day were counted as one unit. Therefore, if even one of these medications was erroneous, that patient-day was counted as erroneous. In the medication-day method, medications were analyzed separately, but a medication that was repeated in several orders on the same day was counted as one medication-day. If a medication was erroneous in one of these orders, then that medication-day was counted as erroneous. Reporting errors per order solved the problem of putting several orders in the same package; however, simultaneously prescribed medications in an order became one unit of analysis. Therefore, if one of these medications was erroneous, then that order was considered erroneous. In the ordered-medication method, each medication in each order was the unit of analysis. Therefore, an erroneous medication in one order did not adversely affect the other ordered medications.

In addition to the error calculation method, the data collection method and review process can affect the error rate [43]. Studies such as Simpson et al [36] that are based on critical or spontaneous reports can detect only a fraction of medication errors [44]. The reason is that these methods are heavily dependent on the individuals and their willingness to share their errors. In hospitals where staff members are afraid of punishment, there will be a lower tendency to openly report critical errors. In the study by Simpson et al [36], the error rate before the educational intervention by a pharmacist was 24.1 per 1000 neonatal days and after the intervention was 5.1 per 1000 neonatal days.

Chart reviews, especially when they are coupled with voluntary reports as in the study conducted by Kaushal et al [7], can detect a higher proportion of prescription errors; the error rate in this study was 5.5 per 100 orders. Direct observation is appropriate for detecting administration errors [44], although it is prone to biases such as the Hawthorne effect [45]. Furthermore, studies like Cordero et al [10] that have reviewed handwritten and electronic medical records have detected a higher rate of medication errors. Such studies have reported the error rate to be as high as 13 per 100 orders.

In our investigation, we reviewed both the handwritten and electronic medical records of orders and nursing charts in both periods. We found the rate of nonintercepted errors to have been 22.7 and 14.5 per 100 orders in the POE and NOE periods respectively. The error rate was 245 per 1000 patient-days in the POE period and 150 per 1000 patient-days in the NOE period.

In summary, methods for calculating and reporting medication errors in neonatal settings are diverse and the results difficult to compare. However, based on all four calculation methods, NOE resulted in a lower rate of nonintercepted dose and frequency medication errors than POE.

### Limitations

This study has a number of limitations. The study was performed in a neonatal setting; therefore, the results may not be generalizable to adults. We selected to study prescribing for the patient group at two points in time because we could not divide patients into two groups—a study group and a control group—in the neonatal ward. Implementation of different medical order entry systems poses a systemic change of the prescription flow in the ward. Moreover, we could not form a control group from another ward of the hospital since the guidelines and dose calculation criteria were very different between the neonatal and other wards.

Since the residents were still in training, their knowledge would be expected to increase over time. This can be a competing explanation of the findings, though previous studies have reported that dose calculation skill among pediatric residents is not related to their experience, grade, level of training, or commitment to recheck their calculated doses [46,47].

Additionally, the care providers knew that they were being studied. Therefore, they might have improved their performance during the study period, which could have led to the Hawthorne effect [45]. This could have affected the results in several ways. Residents knew that one of the purposes of the project was to find the appropriate medical order entry method and to extend this to the other wards of the hospital. It is possible that residents performed better in the NOE period to convince the hospital and university authorities to continue this method and not to return to POE. An opposite attempt by the nurses could also explain the high rate of transcription errors in the NOE period. However, the researchers could not find any evidence of such attempts.

Although the functionality of the decision support system was the same in the two periods, the residents’ trust in the decision support system’s functionality might also have increased over time. This could have led to a higher compliance among the prescribers in the NOE period and could have resulted in better prevention of medication errors in this period. An increase in trust could have happened because of positive experiences of prescribers or other care providers with the system over time and the sharing of those experiences with the others. It could also have happened because of a gradual increase in attention to patient safety among caregivers. However, the influence of these factors would be expected following positive experiences with decision support systems.

### Conclusions

Since physicians' interaction with a dose decision support system is crucial in reducing medication errors, when physicians are resistant to entering orders into the computer and nurses are cooperative and capable of doing so, nurses can perform the order entry with physicians addressing the warnings. This strategy may significantly reduce physicians' resistance and increase their compliance with the system's recommendations. The new order entry method (NOE) can reduce nonintercepted medication dosing errors and increase safety among neonates as effectively as, or even better than, a strictly physician order entry method. However, NOE can increase transcription activities and paper work and add to the complexity of the prescription workflow. An ideal model should reduce physician's data entry workload without increasing the complexity. However, in countries like Iran, where elimination of paper-based medical documentation is not legally possible, POE has no significant advantage over NOE in reducing transcription errors. Therefore, in such settings, NOE could be considered to be a beneficial alternative order entry method.

## Abbreviations

ADE: adverse drug events
CDSS: clinical decision support system
CPOE: computerized physician order entry
EMAC: electronic medication administration chart
EO: electronic order
ERT: error registration table
GFR: glomerular filtration rate
HIS: hospital information system
HWO: handwritten order
NICU: neonatal intensive care unit
NOE: nurse order entry (followed by physician's verification and countersignature)
PBMAC: paper-based medication administration chart
POE: physician order entry
RR: rate ratio
